# Network Architecture for Intelligent Identification of Faults in Rabbit Farm Environment Monitoring Based on a Biological Neural Network Model

**DOI:** 10.1155/2022/6377043

**Published:** 2022-09-10

**Authors:** Hanjie Zhang, Shuqu Qian

**Affiliations:** ^1^Anshun University School of Mathematics and Computer Science, Anshun, Guizhou 561300, China; ^2^Guizhou University of Finance and Economics, Guiyang, Guizhou 550026, China; ^3^University of Sao Paulo, Brazil

## Abstract

Currently, livestock and poultry farming is gradually developing towards modernization and scale, and closed livestock and poultry farms are widely used for poultry feeding management, but at the same time, the farming risks of large-scale farms are increasing. In this paper, based on the study of wireless sensor networks and biological neural network models, the environmental factors that mainly affect the growth of domestic rabbits are analyzed as an example, and the technology is used to design and implement an environmental monitoring system for modern farms. The design of the system is divided into three main parts: hardware design of each node, software design, and upper computer monitoring software design. The hardware part of the system uses coordinator nodes, router nodes, sensor nodes, and control nodes to form a wireless sensor network in the farm, carries out the hardware circuit design of each node, and based on the protocol stack, designs the software program of each node to realize the collection, transmission, and regulation of environmental information in the farm. In the upper computer part, the design and development of the upper computer monitoring software interface are used to complete the real-time display of environmental data, historical query, database storage, and curve drawing, and to design a remote client data query system based on the architecture to realize the query of environmental data of the farm by remote users and to carry out monitoring fault intelligent identification alarm. At the same time, the paper investigates the optimal deployment of wireless sensor network nodes and searches for the optimal location of sensor nodes through an improved biological neural network algorithm to maximize the network coverage and reduce the coverage of blind areas, and conducts simulation experiments with the coverage rate of a rabbit farm as the optimization target.

## 1. Introduction

In the breeding process, livestock and poultry are particularly sensitive to the environment of farm areas (rabbit farms, cattle sheds, pig pens, etc.), and an unsuitable environment can lead to livestock diseases. It is difficult to perceive environmental information in a timely and effective manner by manual means alone. A low level of information in the farming industry can reduce animal welfare and increase animal disease rates [1]. Many farms have substandard feeding environments and insufficient levels of information technology, ignoring the impact of the environment on the growth of livestock, which leads to poor quality of farm products. The requirements of livestock on the growth environment are very strict; in the case of rabbits, for example, rabbits in different growth cycles have different requirements for temperature, humidity, light, and harmful gases. Especially for female rabbits during pregnancy, the requirements for air quality, relative humidity, ventilation, wind speed, and lighting are extremely stringent. An unsuitable environment can lead to low immunity and resistance, slowed growth, and a lower reproduction rate in rabbits. It may even lead to dry and cracked skin and mucous membranes, causing various skin and respiratory diseases, etc., increasing the rate of disease and mortality in rabbits and greatly reducing the yield of farmed products [2]. The merits of the livestock farm environment directly affect the occurrence and spread of livestock and poultry diseases, and the environment around the farm is also closely related to the healthy growth of livestock and poultry and the improvement of product quality. Actually, closed farms need to be insulated in cold weather, but if the air in the poultry house is not smooth, it will cause the index of harmful gases such as carbon dioxide, hydrogen sulfide, and ammonia to exceed the standard, and the temperature and humidity fluctuations in the house will also be large, and the exceedance of these environmental indicators will cause various stress reactions in animals, resulting in lower immunity and triggering various infectious diseases. Therefore, it is necessary to monitor the environmental conditions of farms in real-time and accurately, which not only provides a reference basis for the future regulation of the poultry house environment but also can effectively prevent and control the spread of animal diseases and improve the quality of livestock and poultry products [3].

In this project, we adopt wireless sensor network technology combined with a biological neural network model to design and implement a farm environmental monitoring system with the functions of real-time display, storage, history query, and over-limit alarm for environmental information. The CSPNet architecture is used as a backbone to extract features at three different scales from the rabbit target, and the PANet + architecture is used to generate a feature pyramid in the neck section to aggregate features, which is finally sent to the head module to obtain the output with confidence and box coordinate information using anchored boxes on the feature map. The optimal deployment of wireless sensor network nodes is also studied, and the optimal location of sensor nodes is searched by an improved bio-neural network algorithm to maximize the network coverage and reduce the coverage blind area, and a simulation experiment is conducted with the coverage rate of a rabbit farm as the optimization target.

## 2. Related Work

In general, environmental factors such as light, temperature, humidity, and harmful gas concentration in the farm area are important to the growth of livestock and poultry, so comprehensive monitoring and reasonable control are required. Sensor networks usually have a large number of sensor nodes. How to optimize the deployment of sensor nodes to achieve maximum network coverage, reduce network resource waste, and improve performance is of great importance. The network quality is also a concern.

Air [4] was collected from livestock farms and analyzed for its quality. The results showed that changes in the concentration of carbon dioxide in farms can have different effects on the growth and reproduction of livestock and poultry, etc. Finally, a reasonable mathematical model was proposed based on mathematical modeling to estimate its emission. A powerful chicken house control software package [5] was designed to meet a variety of needs, consisting of a variety of control application modules, which can flexibly regulate the chicken house environment; a wireless sensor detection device for rabbit farm odor was developed [6], which can be used not only to monitor the breeding environment but also to analyze the health status of livestock and poultry-based on odor, health status, etc. To reduce the occurrence of diseases such as foot-and-mouth disease in rabbits, Bumanis et al. [7] prevented the occurrence of diseases by installing wireless sensor networks in their poultry houses to analyze and compare the environmental parameters collected in real-time. In the literature [8], in response to the problem that most current greenhouse monitoring system solutions are limited to small-area temperature control system applications and do not apply their routing functions to extend to large-area applications, a solution design of wireless greenhouse monitoring system based on multi-level routing is proposed to solve the problem of monitoring large-area greenhouses for agricultural use. The multi-parameter recorder of livestock barn designed in literature [9] can monitor a variety of environmental data at the same time, is easy to use, and can save historical data for a long time, but its monitoring range of the environment is small; Long et al. [10] [11] Included the temperature, humidity, atmospheric pressure, and harmful gases in the barn into the monitoring range by deploying the barn environment monitoring system. The system has more sensor nodes, but has not studied the deployment location of sensor nodes; at the same time, the environment monitoring system of livestock and poultry houses based on bus is studied. Compared with the traditional environment monitoring system of livestock houses, the system improves the real-time and reliability of the monitoring network, but the layout of communication lines is more cumbersome, which is not conducive to network updating and easy to be damaged by animals. In the literature [12], a wearable sensor was used to identify the presence of diseases in individual cattle by recognizing their behaviors such as lame walking and head shaking, so that early intervention could be made to reduce the morbidity rate. In the literature [13], a system based on WSN technology was designed to collect and monitor livestock activity data for outbreaks of livestock diseases such as foot-and-mouth disease and avian influenza, and sensors were deployed on farms to diagnose and prevent the occurrence of diseases by comparing the environmental parameters of livestock before and after the disease. Chen and Yang [14] developed a suspected sick rabbit behavior monitoring system using UWB technology. The system uses a hardware platform constructed with an infrared thermometer and indoor precise positioning to monitor the walking trajectory, speed, and direction of rabbits around the clock and monitor whether there are abnormal behaviors such as blind movement and violent advance and retreat to confirm whether the rabbits are suspected to be sick. However, such rabbit identification studs can cause alarm and stress reactions to rabbits when worn, and may also cause discomfort when worn, which reduces animal welfare and is not conducive to welfare farming. Chen [15] designed an automatic monitoring system for sick rabbits' behavior, which monitors rabbits' excretion behavior, records the number of rabbits' excretion, and integrates wireless communication, image processing, and embedded technology to locate suspected sick rabbits with abnormal behavior through algorithms. The images are eventually transmitted to the monitoring center, replacing the traditional human recording method. Weng et al. [16] integrated an individual model of poultry determined in advance and a house regulation model to establish an intelligent monitoring management system for livestock and poultry breeding, which can collect environmental parameters while comprehensively evaluating the optimal solution for temperature regulation under different price costs of feed, fuel, and electricity in the growing environment of chicken houses, calculating the ventilation duration as well as the heating time to reduce capacity costs and expand economic benefits. The spray cooling system developed in the literature [17] effectively reduces the concentration of respirable dust and ammonia in chicken houses by spraying water when the concentration of harmful gases is monitored to exceed the standard, which greatly reduces the exposure of farm workers to poultry dust and stops the occurrence of comprehensive inflammatory diseases in the lungs such as endotoxin.

## 3. The Overall Design of Network Architecture for Intelligent Identification of Environmental Monitoring Faults in Rabbit Farms

### 3.1. A Multiobjective Rabbit Detection Network Based on Biological Neural Networks

**Figure 1:**
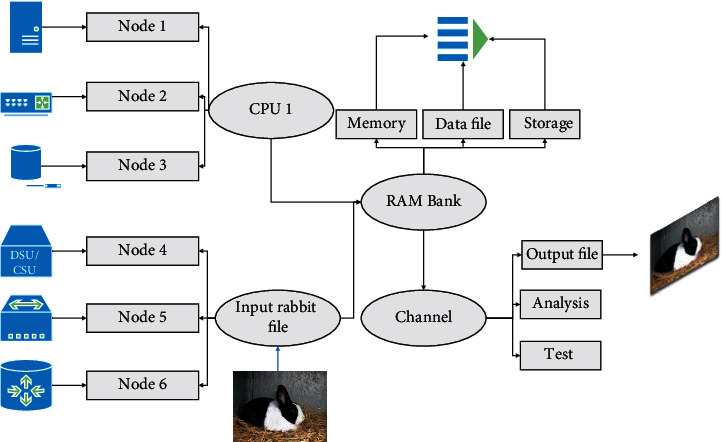
Biological neural network-based multi-objective detection framework for rabbits.

Achieving accurate and fast detection of multi-target domestic rabbits in natural scenes is an important foundation for subsequent multi-target tracking and behavior recognition. To address the problems of the high complexity of existing algorithms, low detection accuracy, and susceptibility to interference by open cattle farm environment and rabbit occlusion, this study proposes an EYOLOv5 algorithm, based on the efficient target detection network YOLOv5, which achieves high-dimensional feature screening and global information aggregation by introducing SA attention module and MSHA module; introduces PANet + to achieve rich scale semantic feature interaction; CIOU loss and weighted DIOU-NMS are used to achieve accurate screening of overlapping target regression frames, to achieve accurate and rapid detection of domestic rabbit targets in natural scenarios and lay the foundation for improving the modernization of domestic rabbit farming [18]. The screening of preselected boxes using weighted DIOU-NMS allows additional attention to the location information of the center points of the bounding boxes for regression, which facilitates the identification of targets with overlapping occlusions. Different degrees of occlusion between rabbit targets are common, and the introduction of LCIoU and weighted DIOU_NMS is more suitable for nonstructured farm rabbit target detection. To achieve accurate and fast detection of rabbit targets in unstructured farm environments, this study proposes a multiobjective detection framework for rabbits in natural scenes based on EYOLOv5, the specific structure of which is shown in Figure 1. The CSPNet architecture is used as the backbone to extract the features of rabbit targets at three different scales, and the PANet+ architecture is used to generate a feature pyramid in the neck part to aggregate the features, which is finally sent to the head module to obtain the output results with confidence and box coordinate information using anchored boxes on the feature map.

Its specific modules are described as follows:

1. Backbone module: based on the CSPNet architecture of YOLOv5, the SA attention module and MSHA module are introduced to achieve the filtering and global information aggregation of high-dimensional features.
2. Neck module: introduction of PANet + module to achieve rich scale semantic features based on the introduction of bridging to achieve mid-level scale feature compensation.
3. Head module: regression of target frames of rabbits of different sizes using 1 × 1 convolution on 3 different scale features of the output, which is the same as YOLOv3 and YOLOv4.
4. Loss module: YOLOv5 uses GIOU Loss as the loss function of the regression frame, and for the unstructured farm environment, this study uses CIOU Loss and weighted DIOU-NMS to achieve accurate screening of overlapping target regression frames.

The main modules of the EYOLOv5 framework include four modules, Conv, Focus, BottleneckCSP, and SPP. Both YOLOv5 and YOLOv4 use CSPNet structure as the backbone to extract rich information features from the input images. The introduction of the CSPNet paradigm module in the backbone network can increase the learning capability of the CNN while reducing computational bottlenecks and memory costs, which help the model maintain accuracy while being lightweight. Specific details of the backbone feature extraction network for EYOLOv5 are as follows:

1. In the case that the original input image is 3 × 640 × 640, the focus structure is entered first, and the four adjacent positions of the image are stacked to focus the W and H dimensional information to the C-channel space, which is conducive to improving the perceptual field of each point and reducing the loss of original information, and the final output feature map is 32 × 320 × 320.
2. Enter the superposition module of Conv + BottleneckCSP, each Conv module (Conv + BN + Relu) has a convolution kernel of size 3 × 3 and stride of 2, which plays the role of downsampling. The BottleneckCSP module implements the extraction of depth features with the same input and output, because the backbone part has 4 CSP modules, the input image is 640 × 640, so the pattern of feature map change is 640-320-160-80-40-20.
3. The SA module is introduced after the last downsampling of Conv to realize the filtering of channel features and spatial features, and then the SPP module is fed to realize the fusion of multi-scale features with different sensory fields.
4. The multi-headed self-attentive module is introduced in BottleneckCSP-T (Transformer) to realize the integration of high-dimensional global features. The final feature maps of layers 5, 8, and 12 (128 × 80 × 80, 256 × 40 × 40, 512 × 20 × 20) are obtained to feed into the subsequent modules to achieve accurate detection of rabbit targets at different scales.

The biological neural network model essentially abstracts the neuron as an RC circuit and views the cell membrane as a capacitance *C*_m_, which is charged by an input current I. Since ions leak slowly through the cell membrane in a nonideal state, the EYOLOv5 model introduces a resistance *R*_m_ into the circuit to reflect the ion diffusion phenomenon in the cell [19]. This segmented nonlinear decreasing strategy improves the global search ability in the early evolutionary stage, and at the same time with the linear adjustment strategy of the acceleration factor, it avoids the premature convergence of the algorithm in the late stage, through which the global search ability and the local search ability can be better balanced, and the convergence speed and accuracy of the algorithm can be improved. Normally, when there is no current input, the membrane potential *V*(*t*) is resting *V*_est_, and only when the output from other neurons is received does the membrane potential *V*(*t*) change. When the voltage at both ends of *C*_m_ exceeds the neuronal voltage threshold *V*_th_, the neuron generates an action potential, followed by a discharge phenomenon of the capacitor, and the neuronal membrane potential returns to the resting state and remains in an absolute resting state for some time. During this time, the membrane potential is not affected by the input current, and the period is called the absolute resting period *t*_ref_. This process graphically simulates the activation of a biological neuron and the delivery of pulses. It is shown in the following equation:

(1) $$\begin{array}{c} C_{m}=\left\{\begin{array}{c} \frac{I\left(t\right)\cdot R}{V\left(t\right)-V_{rest}},V_{rest}<10\\ \frac{dV\left(t\right)}{I\cdot dt},V_{rest}\geq 10. \end{array}\right. \end{array}$$

Since the membrane time, constant indicating the voltage delay time is introduced in the EYOLOv5 model

(2) $$\begin{array}{c} Ut_{ref}=\frac{1}{N}\overset{N}{\underset{j=1}{\sum }}Ut_{i}. \end{array}$$

Thus, the membrane potential *V*(*t*) of the neuron can be given by the following equation.

(3) $$\begin{array}{c} V\left(t\right)=\frac{C_{m}}{\overset{M}{\underset{i=1}{\sum }}Ut_{ref}}+I\left(t\right)R. \end{array}$$

The external input current *I*(*t*) is a weighted sum of the presynaptic neuronal pulse signals. The LIF model simulates the role of excitatory/inhibitory synaptic currents and leakage currents in the neuron by simplifying the various ion channels inside the neuron. The advantage is that it is computationally efficient, requiring only about 5 floating-point operations per millisecond for a single neuron simulation, and the dynamics of the synaptic excitatory/inhibitory currents are well defined, as shown in the following equation.

(4) $$\begin{array}{c} R_{k}=\overset{k}{\underset{i=1}{\sum }}f_{k}\left(w_{k}\right). \end{array}$$

Because synaptic connections primarily carry the process of information transfer between neurons, many researchers have attempted to find biological methods of classification by mimicking the structure of biological neural networks and the mechanisms of synaptic plasticity learning. Studies have shown that synapses can be strengthened or weakened over time, a process known as synaptic plasticity. Synaptic plasticity is believed to be the basis of learning and memory in biological nervous systems. If the neurons at both ends of a synapse are activated at the same time, the connection between them will be enhanced; conversely, if both neurons are not always activated at the same time, the connection between them will be weakened.

In addition, this study utilizes weighted DIOU-NMS for preselected box screening, which can additionally focus on the location information of the center point of the bounding box for regression and facilitate the identification of targets with overlapping occlusions [20]. Different degrees of occlusion between rabbit targets are common, and the introduction of LCIoU and weighted DIOU_NMS is more suitable for nonstructured farm rabbit target detection.

### 3.2. Network Architecture Design for Intelligent Identification of Environmental Fault Monitoring in Rabbit Farms

Many factors such as temperature and humidity, ammonia, and other harmful gas concentration in the livestock growing environment will directly affect the growth condition of livestock. Due to the concentration of livestock, livestock barns are closed, poor air circulation, light is restricted, and toxic and harmful gases in the livestock barn are difficult to be eliminated quickly, all of which will cause adverse effects on livestock growth. In short, the five main factors that affect livestock growth are as follows:

1. *Temperature Influence*. The temperature required by livestock at different stages of growth is different, and too high or too low a temperature can lead to livestock morbidity and even cause the spread of infectious diseases, resulting in the death of livestock. Especially young livestock need to be able to adjust their temperature flexibly because their thermoregulatory mechanisms are incomplete. For example, the optimal temperature range for pregnant rabbits is 18–21°C, for lactating rabbits is 20–22°C, and for lactating rabbits is 29–33°C. When the temperature is at the critical value, it will affect growth and production. Therefore, the temperature must be strictly controlled.
2. *Influence of Humidity*. Air humidity and temperature together affect the growth of livestock. When the temperature in the livestock house is in the normal range, livestock room humidity on livestock growth has no significant impact; but in the high-temperature environment, humidity on livestock will grow adverse effects; when the livestock room humidity is low, livestock will be resistant to decline or even cause disease. The relative humidity of 60%–70%, more suitable for the growth of livestock, in practice can be relaxed to 50%–80%. In addition to livestock houses, silage also needs to strictly control humidity. The moisture content of 60% is appropriate. The leguminous forage moisture content of 60–70% is appropriate; the coarse and hard raw material moisture content of 78–80% is good.
3. *Light Intensity*. Light intensity plays an important role in the growth and production of livestock. The impact on livestock production and growth performance is mainly reflected in the light intensity and light time. Proper light helps promote metabolism, which is beneficial to the healthy reproduction of livestock and good for growth and development. In addition, natural light in ultraviolet light also has the effect of sterilization, the right amount of ultraviolet light can also increase the immunity of livestock, but too much light intensity will reduce their metabolism. Therefore, the intensity of light is one of the factors that must be controlled during the growth of livestock.
4. *Harmful Gases*. The air quality in the farm environment plays an important role in the growth and production process of livestock. Ammonia has a high solubility among harmful gases and is easily dissolved in the mucous membranes of livestock. The body tissues of livestock will be strongly stimulated as a result, which not only makes them susceptible to respiratory diseases but also easily causes necrosis of animal tissues and a series of diseases such as paralysis of the central nervous system.
5. *PH Value*. In the process of livestock word feed, especially silage storage, in addition to temperature and humidity, PH value is also a very important influencing factor. Good-quality silage PH value should be below 4 (3.8–4.2).

**Figure 2:**
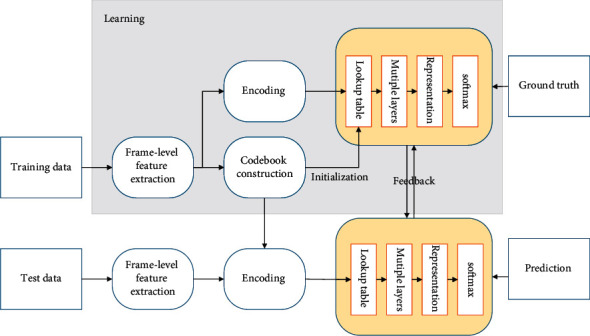
The general structure of the system.

This system takes a rabbit farm as the research object, and each rabbit hutch of the farm is used as an independent control area [21]. The system consists of five parts: ZigBee data acquisition part, PLC control part, centralized control part of the upper computer, remote control, and solar power supply part. The general framework diagram of this control system is shown in Figure 2.

The data collection part consists of coordinator nodes, router nodes, and terminal sensor nodes. Each livestock house has a coordinator node to upload information and send commands; router nodes are used to increase the network coverage, and the network structure in this system is a tree structure for safe and reliable data transmission. An unsuitable environment can lead to problems such as low immunity and resistance, slowed growth, and reduced reproduction rate of rabbits. It may even lead to dry and cracked skin and mucous membranes, causing various skin and respiratory diseases, etc., increasing the disease rate and mortality rate of rabbits and greatly reducing the output rate of farmed products. The terminal node is connected to the sensor, responsible for completing the data collection, using an RF antenna to send the convergence to the coordinator node through the wireless network, and then transmitting the data to the upper computer for centralized display and control through the serial port. After the software analysis and processing of the upper computer, the data can also be transmitted to the PLC control center to further control the action status of each actuator.

The centralized control part of the upper computer mainly adopts the configuration software Configuration King to display and control the status of the system centrally through the configuration king screen, and monitor the safety of the farm and the real-time growth condition of livestock at any time. The main functions of this part include displaying the environmental parameters of the livestock house, the safety condition of the farm, the working status of the system and the setting of important parameters, report generation, and printing, as well as the historical data query function.

On the one hand, the PLC control part receives the problem data sent from the host computer and analyzes the data through the program, then controls the corresponding actuators to make the problem data normal; on the other hand, the PLC itself measures the temperature and humidity, PH value, and other data of the silage warehouse; selects a suitable control strategy; and controls the actuators such as fans and filling pumps through the inverter.

The cell phone remote control part is realized by using the cell phone and TC35 SMS module. GSM SMS module in hardware only needs to connect the upper computer with serial line RS232 and configure the equipment and parameters on Configuration King; then, it can communicate with serial data for remote control. When the farmer needs to control the farm's equipment remotely, he or she only needs to send an SMS to the SIM card number in the SMS module from his or her cell phone [22]. Similarly, when the farmer wants to know the situation on the farm, he or she only needs to use his or her cell phone to send a query SMS to the SIM card number in the GSM SMS module, and the situation on the farm will be sent to the user's cell phone in the form of an SMS.

The design uses an independent solar power supply system, which consists of solar panels, a battery bank, a charge and discharge controller, and an inverter. When there is a cloudy day or night when the solar energy supply is insufficient, the power supply will be switched to the battery pack, and when the battery supply has problems, it will be automatically switched to the general power grid.

## 4. Optimized Deployment of Sensor Nodes for Rabbit Colony Monitoring

Rabbits are generally flexible and mobile in rabbit hutches, making it difficult to monitor their precise activities, which can lead to a large amount of wasted resources if the nodes are not optimally deployed. In this paper, we propose a sensor node optimization algorithm for rabbit hutch distribution, which can significantly improve the application efficiency of sensors and reduce the waste of resources. A wireless sensor network is composed of a large number of sensor nodes deployed in the monitoring area, and its main task is to send the local information data monitored by the sensor nodes to the base station [23]. The optimal coverage of the network is an important issue in the wireless sensor network. Since each sensor node is usually randomly placed in the target area. Reasonably deploy a limited number of sensor nodes in the monitoring area so that their monitoring range covers the monitored target area as much as possible, reduce the overlapping coverage of the target area, and ensure that the network collects information in a timely and comprehensive manner, which is of great significance to reduce the waste of network resources and improve the network performance.

In general, the sensing model of a sensor node is reduced to a model. The commonly used sensing model in the two-dimensional plane is the sensing disc model, i.e., the sensing range of a sensor node is a circular area with the node as the center and the radius, and the value of the sensing radius *B*_*κ*_ is determined by the physical characteristics of the node's sensing unit.

(5) $$\begin{array}{c} UB_{\kappa }=\max_{\kappa }\left(P\right)+\eta \frac{\left(\max_{\kappa }\left(P\right)-\min_{\kappa }\left(P\right)\right)}{dev}. \end{array}$$

**Figure 3:**
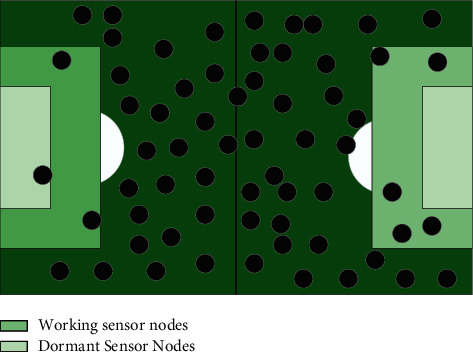
Fence overlay form.

In early coverage studies of wireless sensor networks, the perception model is more often used, but it ignores the influence of the sensors themselves and external physical environment factors and assumes that the sensor nodes are deterministic about the monitoring of events, simplifying the coverage problem. However, in practical applications, due to the influence of noise interference and the node itself, the sensing range of sensor nodes is irregularly circular, and the closer the node is to the node, the better the node's sensing capability for the target. Therefore, the monitoring capability of the sensor node shows uncertainty, and its monitoring model is probabilistically distributed with certain characteristics, and the mathematical expression of the probabilistic perception model is:

(6) $$\begin{array}{c} LB_{\kappa }=m{in}_{\kappa }\left(P\right)-\eta \frac{\left(\max_{\kappa }\left(P\right)-\min_{\kappa }\left(P\right)\right)}{dev}, \end{array}$$

where *η* is the fluctuation value of sensing radius; *de* *v* is a measure of monitoring uncertainty of sensor nodes to represent the dynamic change of sensing radius of sensor nodes; *P* is used to describe the monitoring probability of nodes for events occurring at the target. The main task of a wireless sensor network is to accomplish the monitoring and information acquisition of the target; therefore, while maximizing the network coverage, it is important to take into account the limitations of network energy, number of nodes, and other resources to ensure the provision of reliable monitoring services, and to adjust the location of sensor nodes to enhance the network coverage according to the actual application requirements. For wireless sensor detection of rabbit colonies, the fence coverage method is generally used. The fence coverage is concerned with the monitoring capability of the moving target. When the moving target crosses the monitoring area along a certain trajectory, the sensing range of the sensor nodes should be able to cover the whole moving trajectory of the moving target, which differs from the target (point) coverage problem in that the location of the covered target is uncertain. The curve as shown in Figure 3 is the target moving path.

The advantage of the fence-covering algorithm is that it is simple and easy to implement and there are not many parameters to be adjusted. In the iterative process of the algorithm, it is always hoped that the particles can search the whole space and locate the approximate range of the optimal solution in the early stage, and can improve the convergence speed and search for the global optimal solution in the later stage. The linear decreasing strategy of inertia weights has been proposed. If the neurons at both ends of the synapse are activated at the same time, the connection between them will be enhanced; conversely, if the two neurons are not always activated at the same time, the connection between them will be weakened. This strategy makes the searchability of the algorithm stronger at the beginning, but if the optimal particle is not searched, then with the enhancement of the local search ability at the later stage of the algorithm, the algorithm is easy fall into local extremes and difficult to jump out, which will lead to premature aging of the algorithm [24]. To overcome this drawback and obtain a better optimization effect, this paper improves the algorithm by using a strategy of dynamic adjustment of inertia weights in segments and linear adjustment of acceleration factors in the following equation:

(7) $$\begin{array}{c} \nabla_{b^{t}}J\left(w,b;x_{i}^{*},y\right)=\delta^{l+1}\frac{\partial J\left(w,b\right)}{\partial b_{ij}^{l}}+\lambda \alpha_{ij}^{l}. \end{array}$$

**Figure 4:**
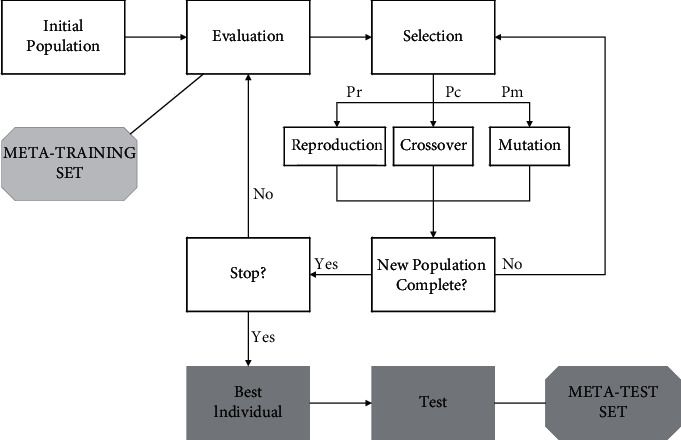
Design flow of the optimal deployment scheme based on rabbit colony monitoring sensor nodes.

The basic idea of this improvement strategy is: by establishing a nonlinear function to dynamically adjust the value of inertia weights *J*(*w*, *b*) so that the pre-evolutionary stage slowly decreases, the particle explores the whole feasible solution space as much as possible with a larger speed to discover a better region; in the post-evolutionary stage, the decreasing trend accelerates, the particle speed *x*_i_^*∗*^ becomes slower and starts to conduct a fine local search. Meanwhile, a linear strategy is used to adjust the value of the acceleration factor*λ* so that the algorithm has a large at the early stage of the search, and the motion of the particle mainly refers to its historical information, and at the later stage of the search by increasing to pay more attention to the population information, so that the particle ensures a certain search speed and avoids premature convergence. The design flow of the optimal deployment scheme of sensor nodes based on rabbit colony monitoring is shown in Figure 4.

This segmented nonlinear decreasing strategy improves the global search ability in the early evolutionary stage, while with the linear adjustment strategy of the acceleration factor, the premature convergence of the algorithm in the late stage is avoided, through which the global search ability and the local search ability can be better balanced, and the convergence speed and accuracy of the algorithm are improved.

## 5. Experimental Verification and Conclusion

### 5.1. Monitoring Sensor Node Optimization Deployment Validation

**Figure 5:**
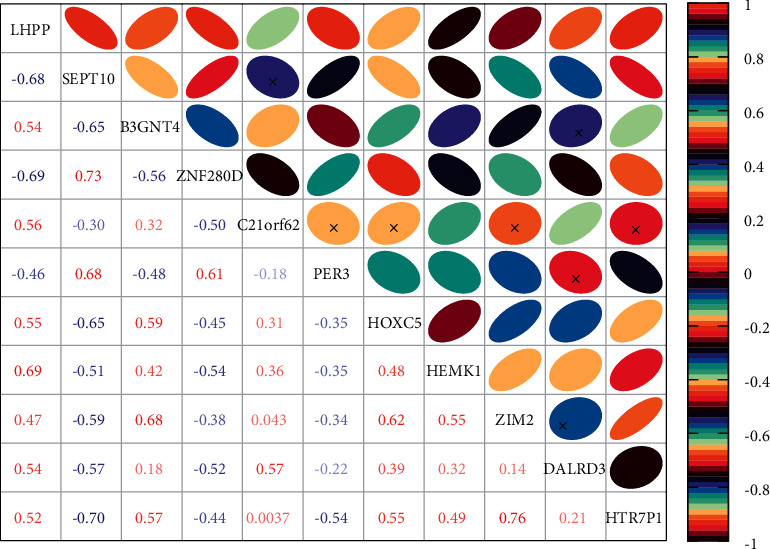
Distribution of nodes after algorithm optimization.

Figure 5 shows the distribution of sensor nodes after the standard algorithm and the algorithm optimization, respectively. By comparison, it can be seen that the distribution of sensor nodes after the standard algorithm optimization has been improved, but there are still some duplicate coverage areas, while the distribution of nodes after the algorithm optimization is more uniform, and there is no duplicate coverage.

**Figure 6:**
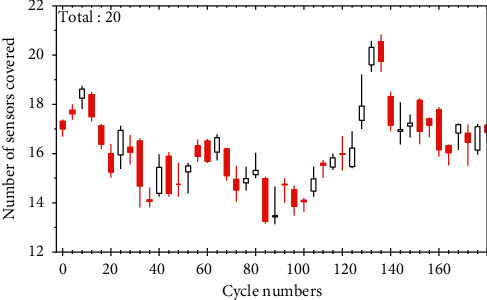
Optimized network coverage graphs.

As shown in Figure 6, the initial coverage of the sensor network is 85%; using the fastest convergence speed of the algorithm, the algorithm has converged to the global optimum at 200 iterations, and finally, the effective coverage of the optimized network can reach 94.3%; at 64 iterations of the algorithm, the optimized effective coverage is higher than the original sensor deployment method. This is because the particles traverse the entire search space at a larger search speed as the blank range slowly decreases in the early stage of the search, which improves the probability of searching for the optimal solution. In contrast, the standard algorithm is used to gradually converge to the optimal solution only when iterating to generations, and the network coverage after 126 iterations is 95.7%. This is because the nonlinear decreasing strategy of inertia weights is used in the algorithm, which makes the global search ability at the beginning of the iteration larger, and the particles can traverse the whole search space at a larger speed to determine the range of the optimal solution; the blank range decreases rapidly at the late iteration, and the particles begin to gradually converge to the optimal region for fine local search. At the same time, with the introduced linear adjustment strategy of the acceleration factor, the particles can still maintain a certain search speed in the late stage of the search, which avoids the premature convergence of the algorithm. Through the simulation results, we can get that the final network coverage of 99.8% is obtained with only 23 sensor nodes deployed, and it is calculated by Equation 6 that at least 41 sensor nodes need to be deployed to achieve this coverage. Compared with the standard algorithm and algorithm, the improved algorithm can optimize the deployment of sensor nodes more effectively, improve the network coverage, and reduce the deployment cost of the network.

### 5.2. Validation of a Biological Neural Network Algorithm for Rabbit Colony Recognition

**Figure 7:**
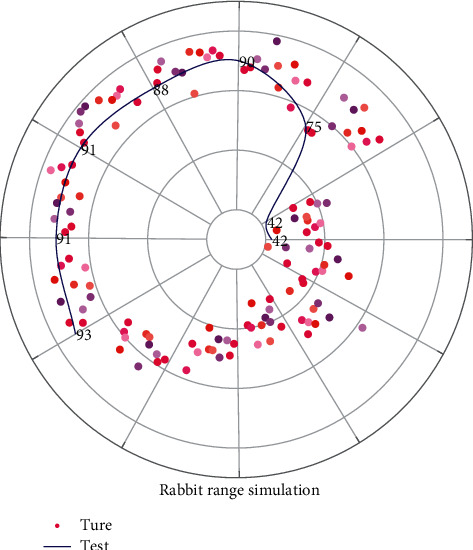
Variation of rabbit group activity recognition accuracy with the number of neurons.

Different from the training method of traditional artificial neuron networks, when training the YOLOv5 network, it is necessary to set a fixed simulation time to deliver the pulse information. In this paper, we set the simulation time of each rabbit activity as 250 ms, of which 200 ms is the learning time and 50 ms is the resting time. During the resting time, the parameters such as membrane potential and synaptic conductance of neurons will decay to the initial value with time to prepare for the learning of the next rabbit activity. Since each sensor node is usually randomly placed in the target area, it is important to reduce the waste of network resources and improve the network performance by how to reasonably deploy a limited number of sensor nodes in the monitored area so that their monitoring range can cover the monitored target area as much as possible and reduce the overlapping coverage of the target area to ensure that the network can collect timely and comprehensive information in the target area. The first set of experiments investigates the effect of network size of SNN on the classification of rabbit population activity dataset when the YOLOv5 algorithm is used. This subsection uses 100, 225, 400, and 625 excitatory neurons to train 60,000 samples in the MNIST training set, 10,000 samples each time, and the whole training set is trained five times to record the variation of the classification accuracy of the network with the number of neurons, as shown in Figure 7.

After 30 iterations, the classification accuracy of the network increases as the number of neurons in the network increases. The classification accuracy is 86.13% for the network with 100 neurons, 88.92% for the network with 225 neurons, 91.01% for the network with 400 neurons, and 91.48% for the network with 625 neurons. Although the classification accuracy of the network is improving with the increase of the number of neurons, the training time of the network is seriously increased because the number of training weights increases from 490000 to 705600 while the number of neurons is increased from 625 to 900, which increases 215600 parameters, so only a network of 625 neurons is trained in this paper. To visualize the features learned by the excitatory neurons, for each excitatory neuron in the 625 scales, the 784-dimensional vector of weights connected to the encoding layer is rearranged into 28 × 28 matrices. The initial weights are random numbers between [0.01, and 0.2]. As the simulation proceeds, the distribution of weights of excitatory neurons gradually converges to their preferred input information.

**Figure 8:**
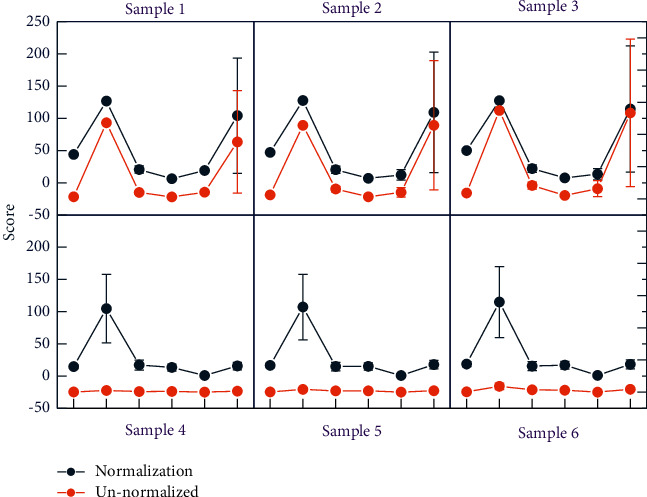
Effect of normalization on the excitation frequency of neurons.

The final MAP of the original network can be seen in Figure 8 as 50.55%, which will be used as a baseline value for subsequent comparison with the calculated value of SN. In the same way, the original mAP of the YOLOv3 network can be obtained as 65.36%. Next, the parameters are extracted from the converted “.h5” format file, the parsed weight file is obtained similarly to the target identification experiment, and the parameters are normalized for the detection experiment. As in the original ANN network, the membrane potential of the final output layer was decoded to obtain specific values, and the prediction frame was drawn on the input test map using the coordinate conversion formula and the confidence calculation formula in the paper. When conducting the first two experiments, the correlation between the activation values and the converted pulse frequency values was high, but if a deeper network was used for the detection task, the whole network would fail because all the neurons in the back layer were not activated and using different normalization methods, the neurons in the back layer of the network had different activation profiles. Without normalization, most of the neurons are under-activated, so they cannot transmit information at all. With only 23 sensor nodes deployed, the final network coverage of 99.8% is obtained, and it is calculated by equation (6) that at least 41 sensor nodes need to be deployed to achieve this coverage. Compared with the standard algorithm and algorithm, the improved algorithm can optimize the deployment of sensor nodes more effectively, improve the coverage of the network, and reduce the deployment cost of the network. After normalizing the parameters using the normalization factor *p*, although some of the neurons are saturated with activation, this is more beneficial to the final result compared to the most inactive state; after normalizing the channels, more neurons are activated, so more information can be transmitted. It helps a lot to improve the accuracy of the results.

## 6. Conclusion

The quality of the environment in livestock farms is an important factor affecting the healthy growth and product quality of livestock and poultry. Scientific and reasonable monitoring and control of the farm environment can not only create a good growth environment for livestock and poultry but is also significant for human health and improving modern production levels. This paper designs a technology-based farm environment intelligent monitoring system, which effectively solves the problems of the existing wired monitoring system, takes rabbits as an example to study the main environmental factors affecting their growth, and selects temperature, humidity, light, and ammonia as the environmental parameters to be monitored by the system. An optimized deployment method of sensor nodes based on an improved algorithm is also proposed to maximize the coverage of the wireless sensor network, which can effectively reduce the blind areas of network coverage and propose an effective optimization solution for the practical application of sensor node deployment in the future. The system is easy to arrange, stable in transmission, and simple in operation. It can realize 24-hour remote monitoring and recording data of livestock and poultry breeding, save time and energy for breeders, improve job satisfaction and efficiency, reasonably manipulate control equipment according to reliable data, and ensure the health and quality safety of livestock and poultry products. The system has been tested in the field, and the test results show that after adding router nodes, the sensor network can effectively increase the communication distance while ensuring reliability, and the system can realize the collection, storage, and display of environmental information and can be alarmed and controlled according to the set value. The system meets the design requirements and can meet the needs of modern farm environmental monitoring intelligence.

Considering the development cost, this system only collects and monitors four types of environmental information, but many environmental factors affect the growth of livestock and poultry, and the monitoring of other environmental information can be added in the future according to the different types of livestock raised in the farm and the actual needs of users, to monitor the environmental quality in the farm more accurately.

## Data Availability

The data used to support the findings of this study can be obtained from the corresponding author upon request.
